# A Layer-Wise Surface Deformation Defect Detection by Convolutional Neural Networks in Laser Powder-Bed Fusion Images

**DOI:** 10.3390/ma15207166

**Published:** 2022-10-14

**Authors:** Muhammad Ayub Ansari, Andrew Crampton, Simon Parkinson

**Affiliations:** School of Computing and Engineering, University of Huddersfield, Huddersfield HD1 3DH, UK

**Keywords:** surface deformation, LPBF, metal additive manufacturing, convolutional neural network, machine learning, deep learning

## Abstract

Surface deformation is a multi-factor, laser powder-bed fusion (LPBF) defect that cannot be avoided entirely using current monitoring systems. Distortion and warping, if left unchecked, can compromise the mechanical and physical properties resulting in a build with an undesired geometry. Increasing dwell time, pre-heating the substrate, and selecting appropriate values for the printing parameters are common ways to combat surface deformation. However, the absence of real-time detection and correction of surface deformation is a crucial LPBF problem. In this work, we propose a novel approach to identifying surface deformation problems from powder-bed images in real time by employing a convolutional neural network-based solution. Identifying surface deformation from powder-bed images is a significant step toward real-time monitoring of LPBF. Thirteen bars, with overhangs, were printed to simulate surface deformation defects naturally. The carefully chosen geometric design overcomes problems relating to unlabelled data by providing both normal and defective examples for the model to train. To improve the quality and robustness of the model, we employed several deep learning techniques such as data augmentation and various model evaluation criteria. Our model is 99% accurate in identifying the surface distortion from powder-bed images.

## 1. Introduction

Recent advancements in additive manufacturing (AM) have revolutionised the manufacturing industry. AM has the potential to reshape the medical [1,2,3] and aerospace [4,5] industries. Laser powder-bed fusion (LPBF) is a prominent additive manufacturing technology [6]. LPBF builds a 3D object from metal powder, layer after layer, by melting a specific area of each metal powder layer. Constructing objects in this manner has several potential benefits over traditional subtractive manufacturing. The most prominent of which are geometrical flexibility [7], rapid prototyping [8], reduction in manufacturing costs [9] and ability to produce complex geometric objects. However, building an object layer by layer can pose many problems. The printing process of LPBF has a unique thermal cycle, and it has been characterised in three parts by Li et al. [10].

1. Rapid heating rate with an abrupt temperature gradient
2. Rapid cooling rate
3. Concurrent melting of the current and previous layer.

The rapid melting of the powder layer due to high energy input (laser power) causes a large temperature gradient. This is followed by rapid cooling of the melt pool due to its small area. Finally, melting the top layer and re-melting previous layers presents a unique thermal cycle. This thermal cycle is very similar to a welding process, and both are incredibly prone to residual stress [11]. If not monitored and controlled, continuous and rapid heating and cooling can result in defects such as lack of fusion, balling, residual stress, voids, cracks, deformation, and warping. Moreover, reducing the printing cost demands the reuse-ability of unfused powder from printing jobs [12]. Surface deformation is a crucial AM defect. It not only results in an undesired geometry of the 3D object but can also halt the printing process if left unchecked. In severe cases, the successive distortion can damage the recoating blade of the printer [10].

Thermal gradients and residual stress are the leading causes of surface deformation defects in LPBF. Distortion and residual stress are impossible to avoid entirely during printing [13]. There are two types of surface deformation known as distortion and warping. Distortion is any deviation in shape, whereas warping is the amount of bend in the final object compared to the original design [14]. For a large, thin-walled, 3D object, the continuous shrinking and expansion of the melt pool, due to repetitive melting and cooling of metal powder layers, causes distortion [15]. Residual stress is the stress that remains after the removal of all external forces. It significantly compromises the mechanical properties and performance of the 3D objects. Sames et al. [16] stated that cracking could occur if the residual stress exceeds the material’s ultimate tensile strength (UTS). The temperature gradient between the current and previous layers is critical in warping and distortion. Acute stress is induced due to surface tension gradients when two fluids are subjected to a temperature difference. This difference pushes the fluid to move from low surface tension (hot region) to high surface tension (cold region) [17]. This means a melted layer on top of a melted layer would have a lower temperature gradient and less chance of surface deformation than a melted layer on top of a substrate (powder layer). Benda and Bourell [18,19] suggested that power transferred to the current powder layer should be selected according to the previous layer, as the power to the current layer might not be sufficient to wet the previous cool layer. Similarly, Rehman et al. [20] studied the meltpool flow to improve the understanding and reliability of the LPBF process.

The effect of different processing parameters on surface deformation is also a keen area of interest for researchers. Li et al. [21] studied the thermal behaviour of the melt pool by varying scan speed and laser power. They found that increasing laser power (100 to 200 W) increased the thermal gradient, and scan speed (50 to 200 mm/s) variations have less effect. Leis et al. [22] also emphasised the importance of optimal values of printing parameters (laser power, laser beam diameter) for excellent surface quality. Liu et al. [23] emphasised the importance of correct energy input on the final quality of fabricated objects. Zaeh et al. [24] observed the surface deformation caused by the heat gradient. Experiments showed that increasing layer thickness from 50 to 70 µm caused minor deformation (−0.015 mm) compared to a decreased layer thickness of 30 µm which caused significant deformation (−0.083 mm). Denlinger et al. [25] studied the effect of dwell time on distortion and residual stress for Inconel 625 and Ti-6Al-4V metal powder. For Inconel 625 metal powder, a high dwell time from 0 to 40 s resulted in decreased residual stress and distortion. Whereas, for Ti-6Al-4V, no or short dwell time decreased the distortion. Cheng et al. [26] studied the thermal stress in the overhang areas of the 3D build. Experiments revealed that the overhang area has more tensile stress, high temperature and significant distortion for ideal values of processing parameters.

The leading cause of distortion and surface deformation, the residual stress, could be avoided by controlling the temperature gradient, printing parameters such as scanning strategies, and mechanical control. Several researchers employed infrared (IR) cameras to monitor the temperature of the melt pool. Berumen et al. and Krauss et al. [27,28] used cameras and photodiode sensors to monitor the melt pool temperature. Klingbeil et al. and Jendrzejewski et al. [29,30] stated that pre-heating the substrate reduced the distortion significantly. Buchbinder et al. [31] examined the pre-heating effect of the aluminium substrate in the selective laser melting (SLM) process. The distortion was avoided entirely when the substrate was pre-heated to 250 ${}^{\circ }$C. Along similar lines, Kempen et al. [32] stated that pre-heating the substrate at 200 ${}^{\circ }$C minimised distortion and produced 99.8% dense 3D objects produced with M2 high-speed steel (HSS). Hauser [33] pointed out that adjusting the laser power per the previous layer’s temperature could reduce part growth near edges, warping, and increase uniform adhesion between the adjacent layers. Mertens et al. [34] stated that pre-heating the powder-bed produced better 3D objects with homogeneous micro-structure properties and were less prone to distortion. Similarly, Ali et al. [35] found that pre-heating the powder-bed at 570 ${}^{\circ }$C reduced the residual stress remarkably. Whereas Dai, K and Shaw, L [36] found that the proper laser scanning pattern changing its direction at 90${}^{\circ }$ on every turn significantly reduced the distortion significantly in nickel metal powder. Similarly, Kruth et al. [37] observed the effect of different scanning strategies on surface deformation. Experiments revealed that an island scanning strategy could be used to combat distortion.

In this work, we propose a Machine Learning (ML) solution to identify surface deformation in real-time using powder-bed images. One of the biggest challenges of ML solutions in AM is the unavailability of labelled data. To overcome this problem, we have designed 13 metal geometries with overhangs. Based on the literature, we ensured a large temperature gradient in the overhang region, sufficient to cause surface deformation defects. The captured data was then used to train a convolution neural network (CNN). CNNs had been widely used to detect defects from powder bed images. Chen et al. [38] used CNNs and object detection algorithms to identify powder spreading problems with an accuracy of 94.38%. Ansari et al. [39] had used CNNs to identify porosity defects from powder bed images. Barile et al. [40] also used CNNs for damage detection during printing from acoustic emissions. They used SqueezeNet CNN and k-Fold cross-validation to achieve 100% accuracy. Though many models exist for machine learning, CNNs have been chosen for their proven ability to work well with image data. Moreover, deep learning-based models i.e., CNNs, are 1.5 times more time efficient than the traditional ML models [41]. Our proposed CNN model is presented as a promising tool to identify surface deformation defects by recognising functional patterns from powder-bed images. The model is 99% accurate in recognising surface-deformed images from standard images.

The remainder of the paper is organised as follows. Section 2 explains the experimental design and methodology. Section 3 presents the results and discussion, and finally, conclusions are presented in Section 4.

## 2. Materials and Methods

**Figure 1:**
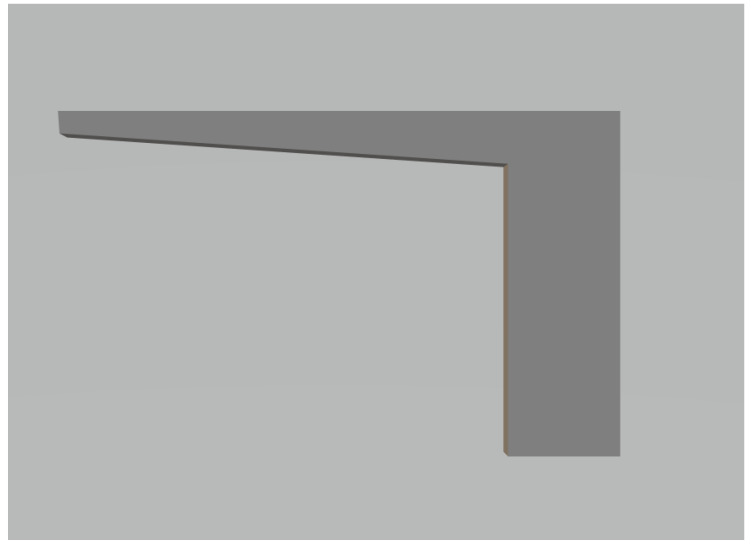
CAD design of the test specimen.

To study the surface deformation effects in LPBF, we designed and printed 13 bar-like geometries. The design of a bar is shown in Figure 1. The choice of test specimen geometry was carefully selected. The test specimens were intentionally designed with an overhang to cause surface deformation in a naturally occurring way. Overhang, or down-facing geometry, is a common design feature and a challenging task to print. Usually, creating a support for an overhang is an important design aspect. However, it is not always possible to support overhangs in 3D objects. It is not our intention to either study or evaluate various design constraints but rather to determine the extent to which machine learning can detect naturally occurring deformation. Overhang printing, without support, causes a large thermal gradient to occur, resulting in the deformation of the 3D object. Therefore, we can naturally cause deformation in the test specimens by omitting the overhang support during the printing process and capturing good and bad data examples for model training.

**Figure 2:**
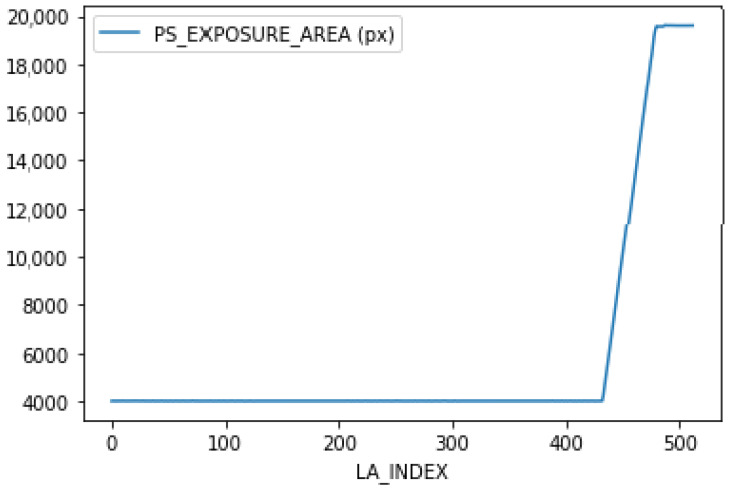
Area of a layer (No of pixels) exposed to laser power vs layer numbers of test bar1.

The test specimens have a bar-like base designed to be printed without surface deformation. Overhang is designed in the final section of the geometry. The overhang part is printed on top of a metal powder substrate, causing a temperature gradient, which results in surface deformation. The overhang area is slowly increased through the build, where the distortion effect will become more and more prominent. The reason for not creating a bar-like overhang is to ensure the temperature gradient. A bar-like overhang would have distortion only in a few layers; as the upper layers would be printed on melted layers. The area of the overhang region slowly increases in size from layer number 433 to 491. This was confirmed by the “Parts Statistics” file from the EOS printer, which contained the information of the different jobs, their respective IDs, layer information, layer height, job name, and the number of pixels exposed to the laser per layer. A line plot, shown in Figure 2, between the area of a layer in pixel exposed to laser power vs layer number, provided important insight for defect identification. It was observed that the first 433 layers were correctly printed as the area of layers exposed to the laser was consistent. However, after layer 433, the overhang part was printed on the powder substrate. Surface deformation was observed from layer 433 to layer 511. The area of the last 20 layers was consistent to limit the successive deformation. However, the distortion in the test specimen was left unchecked for too long and resulted in a re-coater problem.

The layers from 0–433 had a small surface area compared to the layers from 433 to 511. It was essential to ensure that this did not influence the model into using the surface area to distinguish between normal and distorted images. The last 20 layers were designed to overcome this problem by ensuring that the surface area of layers 491 to 511 was close in size to the surface area of the deformed layers. The successive distortion from the previous layers also affected the last layers.

### 2.1. Instrumentation and Data Capture from EOS Printers

The objects were printed at AMEXCI AB, Karlskoga, Sweden, using AlSi10Mg metal powder and default printer settings on an EOS M290 machine (AMEXCI AB, Karlskoga, Sweden). The experiment aimed to induce surface deformation using geometry variation. A uniform layer thickness of 30 µm was used. The laser power of 370 W, hatch distance of 0.19 mm, and a scan speed of 1300 m/s were used in the printer setup. The final 3D objects were of height 15,330 µm and contained 511 layers altogether. The EOS printer was equipped with cameras and sensors that photographed each layer before and after laser exposure.

**Figure 3:**
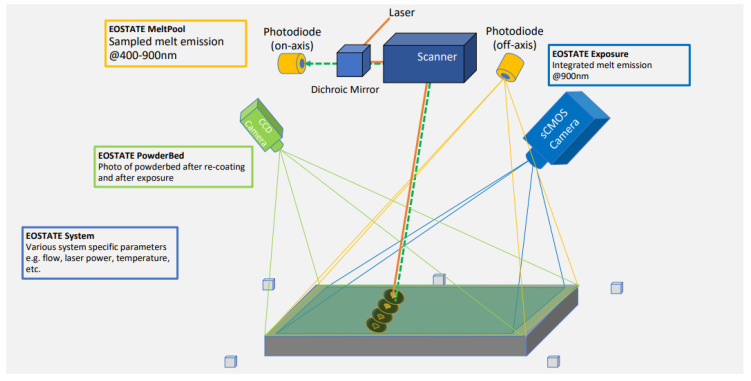
An overview of EOSTATE monitoring suite.

EOS printers are equipped with the EOSTATE suite, which consists of four monitoring systems. These include EOSTATE Exposure OT, EOSTATE Meltpool, EOSTATE System and EOSTATE powder-bed supervision. An overview of EOSTATE Suite is shown in Figure 3. Each monitoring system in the EOSTATE Suite is designed to monitor certain specific conditions. A combined analysis of multiple monitoring systems enables an indepth understanding of process deviations and abnormalities. The recorded data from the monitoring systems will contribute toward closed-loop monitoring, decreasing the need for post-build quality inspections such as XCT or Cross-Sectional analysis. EOSTATE Meltpool is the oldest and most commonly used monitoring, consisting of two photodiodes, one off-the-axis and one on-the-axis. An on-axis photodiode is installed in the laser path using a semi-transparent mirror. The on-axis photodiode records the melt pool radiation intensity around the small region of the melt pool. In contrast, the off-axis photodiode is directly installed on the roof of the printer and records the melt pool radiation of the whole build plate. The signal data, recorded by the two photodiodes, are stored on a separate PC along with the x-y coordinates of the laser scanner path, laser exposure type and duration.

The latest monitoring process, EOSTATE Exposure OT, consists of an sCMOS-based camera to capture high-quality and high-resolution near-infrared (NIR) images. The camera is mounted on the printer’s top and is covered with a neutral density filter to capture the entire platform in a narrow infrared wavelength. The camera’s spatial resolution is 125 µm per pixel, and the resulting images are 2560×2160 pixels in size. The brightness in the OT pictures represents the amount of heat transfer over time. More time exposure to a high-energy laser beam would appear brighter on the OT image. These OT images were used as a benchmark in labelling the data set. The OT monitoring system produced two data files, apart from the raw images. The “List of indications” file contained extreme cold or hot spot information. In comparison, the “Part statistics” file recorded information about the area of each layer exposed to laser power, layer numbers, and the height of the object after each layer’s job ID.

The EOSTATE system records the values of oxygen level, laser power monitoring, gas flow, humidity in the chamber, temperature, layer time and layer thickness. Finally, the EOSTATE powder-bed takes pictures of the build platform before and after laser exposure. The powder-bed images were labelled with the help of OT images. The CNN is trained on powder-bed images to detect surface deformation.

### 2.2. Data Labelling

**Figure 4:**
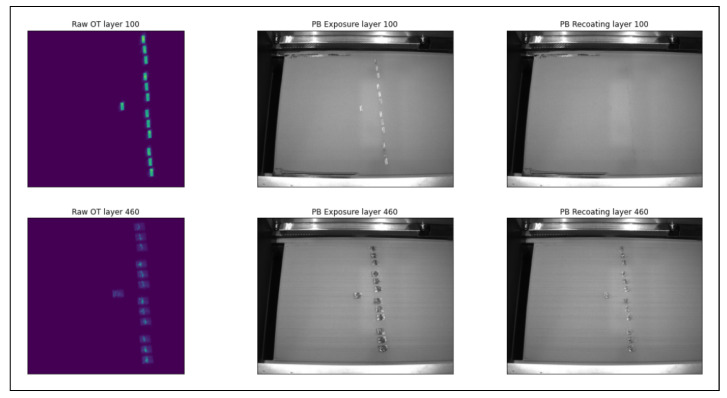
Raw OT image, PB image after laser exposure and PB image after re-coating.

Identifying surface deformation, or any LPBF defects, requires the input of domain experts. However, with the monitoring capabilities of the EOS printers, various abnormalities in the captured data can assist in identifying surface deformation defects. The current data sensing can help machine learning, especially in labelling powder-bed images. It is arduous to identify surface deformation defects from powder-bed images. However, a combined analysis of the OT pictures and powder-bed fusion pictures before and after laser exposure can indicate surface deformation defects. The colour on the OT pictures represents the amount of heat in a particular region. The OT pictures show a bright yellow spot for the correctly printed layers, whereas deformed layers appear cold (blue) on the OT image, and their surface area is also more spread out than usual. The deformed layers were printed on top of the cold powder substrate. Massive temperature gradients resulted in an unstable melt pool, resulting in the deformation of the layer. The deformed layer also appears on the powder-bed images after re-coating. Due to the increase in shape and area of the deformed layer, the next powder re-coating layer can not cover the deformed printed layer; thus, the appearance of the previous layer on the powder bed after re-coating is a clear sign of surface deformation. A comparison between the OT images, the powder-bed image after laser exposure and the powder-bed image after re-coating is shown side-by-side for two layers, layer 100 and layer 460, in Figure 4.

**Figure 5:**
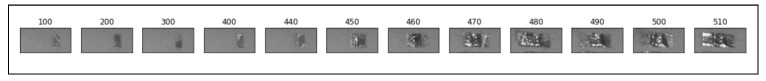
Picture of bar1 at various layers during printing. The layer number is displayed on top of each layer image.

Layer 100 was printed normally while layer 460 shows surface deformation. The OT image shows bright spots on the printed portion of layer 100 in the top left corner of the image. After re-coating in the top right corner, the powder in the powder-bed image entirely covered the previous layer. On the other hand, the OT picture of layer 460 in the bottom left corner shows cold (blue) spots, and the corresponding powder re-coater layer shows the previous layer. So, in general, cold blue spots on OT and a blurry appearance of 3D object slices on powder-bed pictures, especially after re-coating, are clear indications of surface deformation. Labelling the LPBF images is one of the most pivotal and challenging tasks. Correct labelling of images requires an in-depth understanding of how surface deformation occurs and how it appears on the images. Additionally, sensor data information is vital in assigning the correct tag to the images. Figure 5 shows bar1 at various stages (layers) during the printing process. The surface deformations were observed after layer 433 and increased gradually per the designed overhang geometry. The clear observation of surface deformation and the sensor files’ data helped label the images. The images corresponding to layer numbers 1 to 432 were classified as normal. Whereas layer images from 433 to 511 were labelled as defective images showing surface deformation.

### 2.3. Data Engineering

**Figure 6:**
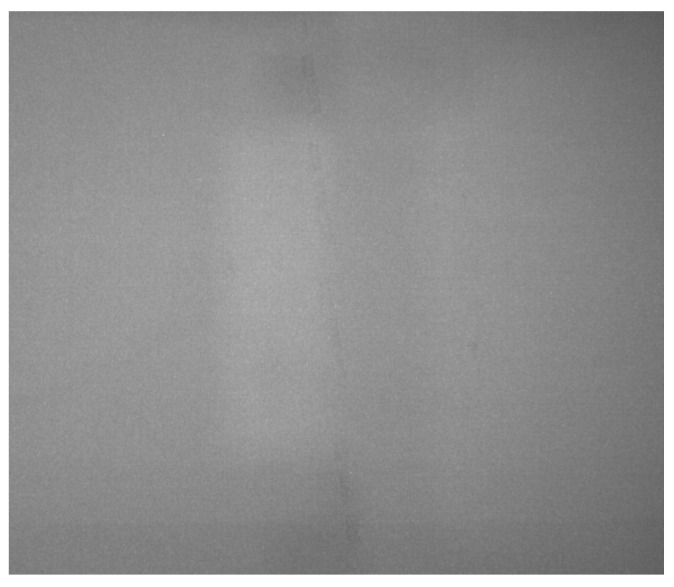
The powder-bed image of layer 20 before laser exposure.

**Figure 7:**
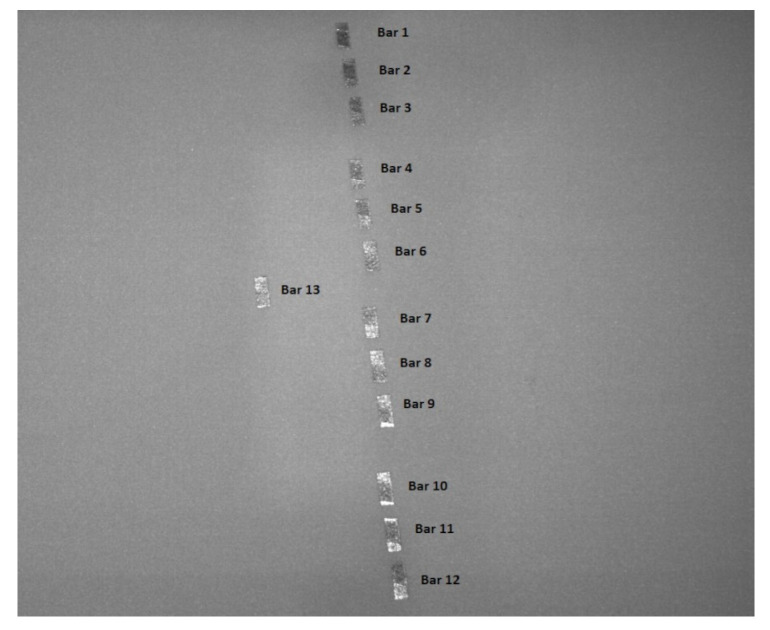
The powder-bed image of layer 20 after laser exposure.

The image dataset of 1022 powder-bed images consisted of 511 before and 511 after laser exposure. The 13 bars, each 115mm in height, resulted in 7.5 gigabytes of data. Amongst them, the powder-bed pictures were the most important. A sample image from the powder-bed both before and after laser exposure, at layer number 20, is shown in Figure 6 and Figure 7, respectively. The powder-bed images were 1280 pixels wide and 1024 pixels in height. The region of interest (ROI) was selected, and the bars were cropped from the whole image. The bars, as shown in Figure 7, were not in line with each other. This presented a challenge in cropping out the bars. Four large chunks were cropped out from the images consisting of three adjacent bars. For instance, the first large cropped picture consisted of bar1, bar2 and bar3. Similarly, other cropped images consisted of three adjacent bars. These large, cropped images were rotated slightly to the right at an angle of −7 degrees to facilitate the cropping of the bars. Each large cropped image was further split into three equal parts containing one bar. The final image sizes were 80×120 pixels. The images were converted into grey-scale from colour-scale and reshaped. The final image data set shape consisted of the number of samples in the data set, the width of the image, the height of the image and the channels in the images. Since all images were converted to grey-scale, the final images had: channel = 1, width = 120, height = 80 and samples = 5832. It is worth mentioning that the final images represented 486 layers of the total 511 layers of each test bar. The first 25 layers were discarded as the initial layer images are always captured in poor conditions due to the reflection from the build plate. The final image data set was converted to a float data type from unit8 and normalised by dividing each pixel value by 255. The resulting normalised image’s pixels were 0 to 1, compared to 0 to 255.

### 2.4. Data Set Augmentation and Selection

Class imbalance is one of the most common problems in data-driven solutions and can result in biased classifiers if not dealt with effectively. The data set was highly imbalanced, with only 15% of the images showing a surface deformation defect. We experimented with both balanced and imbalanced data sets to demonstrate the effect of class imbalance. Due to the overhang geometry, the surface deformation defect becomes too large too soon in the experiments. It not only compromises the test specimens but also physically damages the printer. Therefore, identifying the surface deformation earlier is critical. We sampled two data sets as shown in Table 1. The original imbalanced data set consisted of 414 normal and 72 defective images. The re-sampled balanced data set consisted of 239 normal images from layers 201 to 439 and only 14 defective images from layers 440 to 453 showing the early signs of surface deformation. The intuition behind selecting a minimal number of defective images is to identify the geometric distortion at an early stage and stop its propagation as soon as possible. This is a practical and realistic approach. However, it widens the class imbalance drastically. The defective images are only 0.055% of the total data set; therefore, it is essential to reduce the huge class inequality. The minority class (defected images) was over-sampled using data augmentation methods to combat the class imbalance problem. The data augmentation methods used in the experiments are shown in Table 2. Data augmentation is a robust way to combat class imbalance. The horizontal flip reverses the rows of pixels, and the vertical flip reverses the columns of pixels in the image. The width and height shift move the image pixels in one direction while keeping the same image size. The empty spaces created during the shifting were filled by copying the nearest pixels. Moreover, an angle rotation of 10${}^{\circ }$ was also used to sample more defective images. Angle rotation shifts the entire image as if it was captured from a different angle. The empty spaces created by the rotation were filled with nearby pixels.

### 2.5. Convolutional Neural Network (CNN)

The extraction of valuable features from high-dimensional data, captured via sensors and optical camera images, is a significant challenge that requires intensive computational and human resources. There is no direct, simple mapping that can transform this high-dimensional data into a helpful output, able to clearly distinguish between a defected and a non-defected surface deformation image. In their most basic form, the images are simple two-dimensional grids of numbers. However, convolutional neural networks have a proven ability to automatically extract localised, spatial information from these images in a way that can then be used to discriminate between one image type and another; they are also known for their ability to handle big data sets. The remarkable ability of CNNs to optimally down-sample the extracted feature set via various pooling strategies (pooling layers) makes them both time and memory efficient. As we are working with images taken from the powder-bed layer, CNNs have been chosen for their ability to automatically extract features, learn key spatial patterns and predict surface deformation defects in real-time. For clarity, a brief explanation of the important aspects of CNNs is provided below.

CNNs consist of three unique layer types: a convolutional layer, a pooling layer and a fully connected layer. Like many machine learning algorithms, some parameter values are set during the training process (model parameters), while others need to be selected before training (hyper-parameters). The model-parameters and hyper-parameters associated with CNNs, are shown in Table 3. The convolutional (or feature extraction) layer is the most important layer of a CNN. It consists of several kernels (basic image filters) that perform linear, element-wise operations on the input image to extract spatial features - creating what is called a feature map. This process is repeated with different kernels and results in an accumulated feature map. Each kernel is chosen for its ability to extract different types of features. Typically, the pixel size of these kernels are 2×2, 3×3, 5×5 etc. The larger kernel sizes result in smaller feature maps and vice versa.

Another necessary convolutional operation is the stride, which is the difference between two successive kernel positions. For a chosen filter size, passing over the image with a chosen stride length, it may be necessary to introduce padding at the image boundary—particularly if the size of the feature map is required to match that of the original image. A popular approach is zero-padding, which introduces zero values at the edges of the images. The number of kernels, kernel size, stride length and padding values are the critical convolutional hyper-parameters, and their values influence the generalised training capability of the CNN model.

The output of the convolution layer is passed through a non-linear activation function, such as sigmoid, tanh, relu, to construct the feature map. The feature map is then passed to a pooling layer responsible for down-sampling the feature map. There are two main types of pooling operation: max pooling and average pooling. Max pooling, for example, only keeps the maximum value from a chosen local neighbourhood of values in the pooling layer. For example, if only the maximum value in each local 2×2 region is kept, the feature map can be reduced by a factor of 4.

The process of combining convolution and pooling layers is repeated until a satisfactory performance of the model is obtained. The two-dimensional feature map from the final pooling layer is passed to a fully-connected layer that flattens it down to a one-dimensional array of feature values which are then passed to the dense feed-forward model. The layers in the dense model are trained on this flattened feature map, and the layer weights are adjusted optimally to enable the network to distinguish between the different classes in the input layer. The most common activation function in the dense network’s layers is the Relu function. However, the final layer’s activation function is chosen depending on the type of output decision required (i.e., binary, probabilistic). The training of the CNN model is the process of finding the best values for the parameters such as kernels, weights of dense units, and learning rate. The training process aims to minimise the difference between the network’s predicted labels and the ground truth labels by employing optimisation learning algorithms such as Gradient Descent, Stochastic Gradient Descent, ADAM, and RMSProp for example.

Model architecture is a vital hyper-parameter to tune, and selecting appropriate values is essential. In this study, we propose three different convolutional architectures. The simplest CNN, model1, consists of one convolution, one max-pooling and one dense layer. Model2 has two convolutions, two max-pooling and two dense layers. In comparison, model3 has three convolutional, three max-pooling and two dense layers. The hyperparameters of the models were selected heuristically to acquire the maximum accuracy of the model. The parameters of each model are given in the following Table 4.

### 2.6. Optimisation Algorithm

Machine learning optimisers are the algorithms that set/update the weights and other learning parameters of the neural networks, such as learning rate and batch size for example, to minimise the loss function. We selected the ADAM optimiser, and all the models were optimised using the ADAM optimisation algorithm with default values of:

$$\alpha =0.001,\;\;\beta_{1}=0.9,\;\;\beta_{2}=0.999\;\mathrm{and}\;\epsilon =10^{-8}.$$

ADAM is memory efficient and based on two well-established optimisation algorithms, AdaGrad and RMSProp. It combines AdaGrad’s ability to handle sparse gradients and RMSProp’s strength in dealing with non-stationary objective functions [42]. The ADAM algorithm converges slowly with a small step size to achieve the global, minimum value of the loss function. The parameters were updated as follows:

(1) $$\theta_{t+1}=\theta_{t}-\left\{\frac{\eta *\hat{m_{t}}}{\sqrt{\hat{v_{t}}}+\epsilon }\right\}.$$

where $\hat{m_{t}}$ and $\hat{v_{t}}$ are given by

(2) $$\hat{m_{t}}=\frac{m_{t}}{1-\beta_{1}^{t}}$$

(3) $$\hat{v_{t}}=\frac{v_{t}}{1-\beta_{2}^{t}}$$

Here $\hat{m_{t}}$ is the mean of the first momentum, and $\hat{v_{t}}$ is the variance of the second momentum. The ADAM algorithm is immune to high variance and vanishing learning rate. It converges quickly and speedily but requires high computational resources.

We employed “categorical loss” as the loss function. Other default values in the experiments were the activation function, which was set as Relu and accuracy as the evaluation metric. All the models were trained using early stopping on the validation set accuracy with a patience value of 5.

## 3. Results and Discussion

The experiments were carried out on an Intel core i7 machine with 24 gigabytes (GB) of RAM and 4GB of NVIDIA Geforce GTX 1050 graphical processing unit (GPU). Python 3.7.0 (PSF, Wilmington, DE, USA) was used along with Tensor-flow 2.7.0, Keras, Pandas, Matplotlib and SK-Learn for the experiments.

The performance of CNNs depends on various factors. Apart from having balanced data, ensuring a correct proportion of the two classes in both the training and testing data is also crucial. A stratified train/test split was employed to ensure the same proportion of classes in both training and testing data across all the data sets. The data sets were split into 70% training and 30% testing samples. However, the training data set was further split into 70% training data and 30% validation data during the training process. The test data set was kept separate; the models are never exposed to the testing data set during model training. The number of defective and normal images across training and testing data sets are shown in Table 5. The table demonstrates that the train and test sample sets have the same proportion of both normal and defective classes in training and testing data.

**Figure 8:**
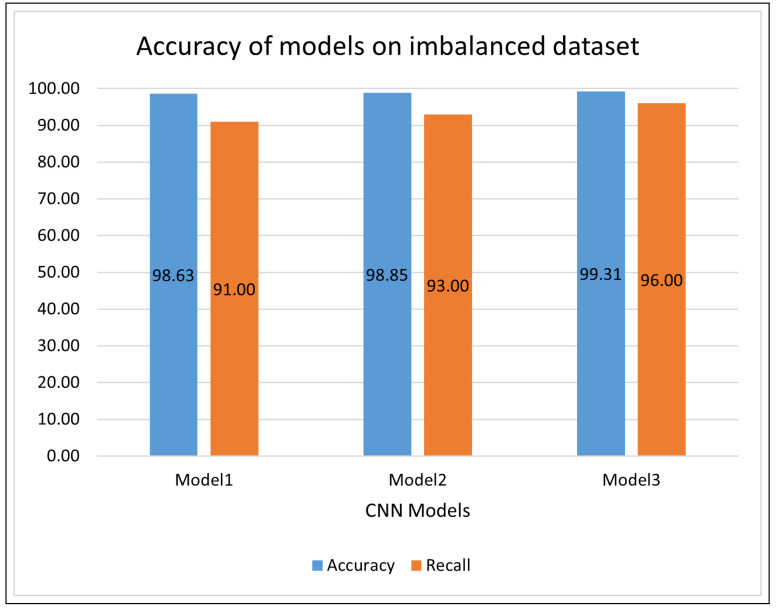
Accuracy of models on the data sets.

Another challenge in differentiating the distorted images from the normal images is the lack of availability of any useful, pre-trained CNN models. All of the well-established pre-trained models, such as VGG [43], AlexNet [44], DenseNet [45], Inception [46], were trained on the MNIST, MS Coco, CIFAR-10, data sets. These data sets are very different to LPBF images in terms of the features learned by these models. As such, these pre-learnt models cannot be transferred over to the LPBF problem. Training a CNN model on a novel data set from scratch is challenging and requires extensive experimentation to find the optimum hyper-parameters. One of the important decisions in training the CNN from scratch is the model’s architecture. Following the Occam’s razor [47] principle, we chose to start the experimentation using a simple CNN architecture consisting of only one convolution layer and one pooling layer and then gradually increased the layers to produce more complex models. The results of all the models on the original imbalanced data sets are shown in Figure 8.

The original, complete data set was highly imbalanced and contained only about 15% distorted images. All three models performed exceptionally well on it and achieved excellent accuracy. It was observed that the simple model1’s accuracy was slightly less compared to the model2 and model3. Model1, with just one convolution layer and one pooling layer, achieved an accuracy of 98.63% on the original, complete, imbalanced data set. The model1 has only one convolutional layer and one pooling layer in the feature extractor part of CNN. More layers in feature extraction extract more refined and decisive features. Moreover, a smaller number of pooling layers results in a bigger feature map and therefore affects the model’s decision-making. That is why the model1’s accuracy was less than model2 and model3. Whereas the more complex models, model2 and model3, with two and three convolutional and pooling layers, respectively, achieved an accuracy of 98.85% and 99.31%. The results of the model3 were superior to those for model1 and model2. Model3 achieved an excellent accuracy of 99.31% and outperformed model1 and model2. The excellent accuracy of the model3 implies that the more complex CNN architecture produced a more decisive feature map. In the rest of the paper, model3 will be referred to as the model.

**Figure 9:**
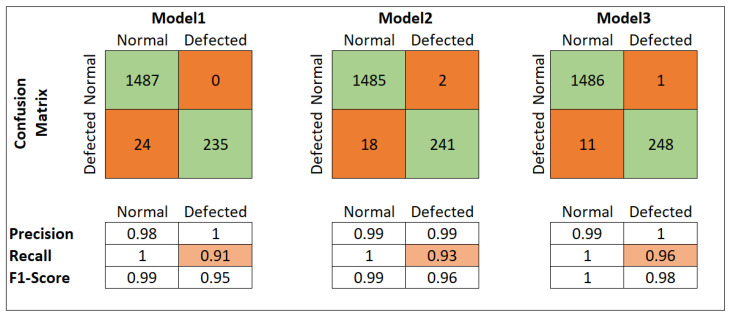
Confusion matrices of models on all data sets.

Accuracy can be a misleading evaluation metric, especially when calculated on imbalanced data. Therefore, we constructed a confusion matrix for an in-depth evaluation of our models. The confusion matrix of the different models on the original imbalanced data set is shown in Figure 9 and demonstrates their actual performance. In the presented confusion matrix:

1. True positive: The total number of surface deformed (defected) images correctly predicted by the model.
2. True negative: The total number of normal images correctly predicted by the model.

In binary classification problems, the number of false-positive and false-negative predictions play a decisive role in model evaluation. Depending upon the problem, the tolerance to one is severely less when compared to the other. For instance, in the binary classification of the COVID infection test, a false negative (person infected with COVID but wrongly predicted as healthy) is more damaging than a false positive (a person without COVID but wrongly predicted as infected). Similarly, in surface deformation defect identification, a false negative is more damaging as it will build the 3D object with an inaccurate geometry. The precision and recall measures can assist in a model’s evaluation concerning false positive and false negative rates from the confusion matrix. Precision and recall are given by,

(4) $$\mathrm{Precision}=\frac{\mathrm{TP}}{\mathrm{TP}+\mathrm{FP}}$$

(5) $$\mathrm{Recall}=\frac{\mathrm{TP}}{\mathrm{TP}+\mathrm{FN}}$$

Here, TN is the true negative, and TP is the true positive. False positives and negatives are denoted by FP and FN, respectively. Precision is the measure of positive cases correctly predicted by the model out of all the positive cases. In contrast, recall measures the correct prediction of the negative class. Accuracy measures all correctly predicted images as a proportion of the total number of images in the test set.

Due to a high imbalance among the data set, model1 failed to train correctly, mainly due to its simplistic architecture. It classified most of the instances in the test data set and acquired a good accuracy but failed to distinguish between defected and normal images decisively. Because of the high imbalance, the model correctly identified the majority class and attained remarkable accuracy, but it failed to identify the crucial defected images. It misclassified 24 defective images as normal images. This is a high number of missclassification of an already under-represented class. That is why its recall for defective images was 91%.

The model2 and model3 with more convolutional and max-pooling layers achieved a better understanding of the data set. Apart from their excellent accuracy, model2 and model3 achieved a recall of 93% and 96%, respectively. Accuracy is a good evaluation metric but misleading for an imbalanced data set. Although all three model architectures achieved excellent accuracy, there is little difference. However, there is a significant increase in model recall with increased feature extraction layers, as shown in Figure 8. The recall measures of model1, model2 and model3 were 91%, 93% and 96%, respectively. Model3 achieved the best recall of 96% with an accuracy of 99.31%. It successfully identifies defective and normal images with only a few miss-classifications, as shown in the confusion matrix in Figure 9. The better performance of the model3, when compared to model1 and model2, is due to its denser feature extraction architecture. It extracted more decisive and powerful features from the input images and drastically down-sampled them to have only a few distinguishable features. The number of trainable features of model3 was 136,538 compared to 1,475,842 for the model1 and 230,730 for model2.

**Figure 10:**
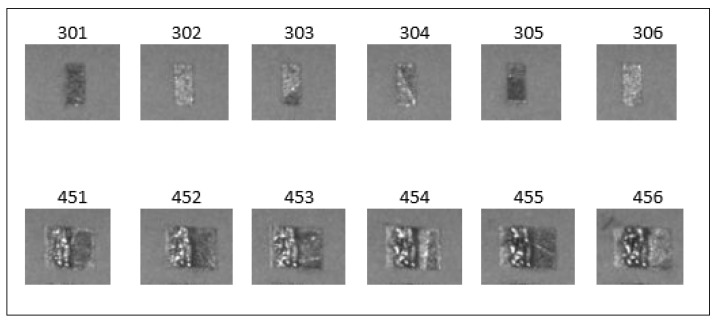
The difference in the shape of the test specimen at different layers.

Surface deformation defects in the experiments were continuous and increased rapidly. Because of this, the difference in the shape of normal and defective images was very prominent. The difference in the shape of the test specimen at different layers on powder images is shown in Figure 10. The shape of the test specimen in layer number 301 to 306 (normal images) is different from the test specimen’s shape in layer number 451 to 456 (defected images). The test specimen appeared as small, compact, rectangular-shaped bars in layers 301 to 306. At layer numbers 451 to 456, its shape appeared larger, more squared and irregular.

**Figure 11:**
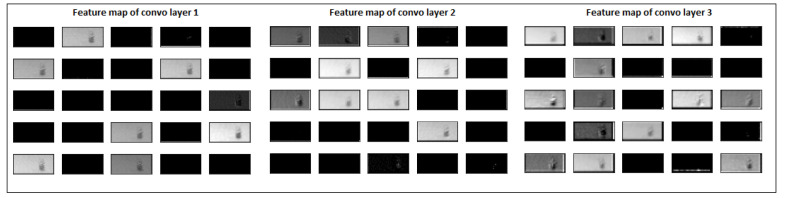
Feature maps of normal layer image 300 after each convolutional layer.

The massive shape difference between the defective and normal images could hinder a model’s training. Instead of extracting distinguishable features for surface deformation, the CNN model might be learning the shape/size difference. We decided to visualise the internal learning of hidden convolutional layers of our model to verify and better understand our model’s actual learning. The feature maps of all three convolutional layers were extracted, re-scaled back to images and visualised to see how the model processed the input images. A normal image of cube1 at layer 300 was passed through the model, and each convolutional layer’s first 25 feature maps are visualised in Figure 11.

**Figure 12:**
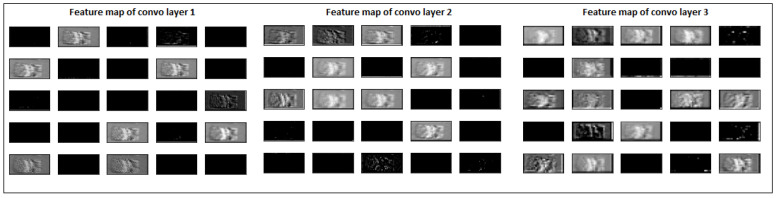
Feature maps of defective layer image 500 after each convolutional layer.

At the same time, a defective image at layer 500 was passed through the model, and the model’s feature map of convolution layers was extracted. It is shown in Figure 12. It is evident from Figure 11 and Figure 12 that the model’s prominent features are influenced by the size of deformation. The first convolutional layer extracted more generalised features such as edges and lines. In comparison, deeper convolutional layers extract high-level, abstract, and problem-specific features. Although the model is highly accurate in differentiating deformed images, there is room for the model’s learning. From a practical point of view, the model’s current learning might not identify surface deformation at an early stage and identify it only when it becomes too large.

**Figure 13:**
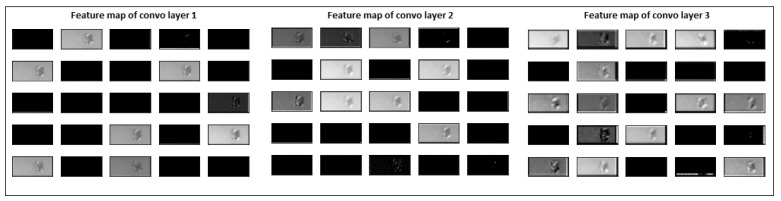
Feature maps of defective layer image 440 after each convolutional layer.

**Figure 14:**
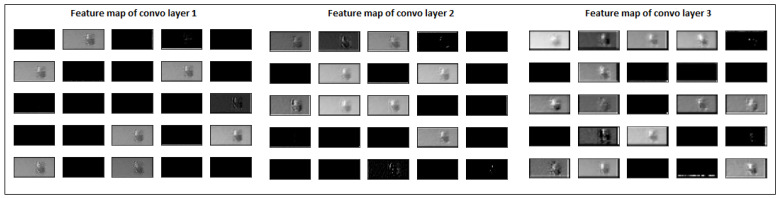
Feature maps of defective layer image 450 after each convolutional layer.

To overcome this problem, we sampled a second data set with many normal images and just the initial 14 layers of images of surface deformation to train the model on a more practical and realistic problem. This will ensure that both normal and defective examples have almost similar surface area or shape on the powder bed images. The feature maps of our model for input defective images from layer 440 and 445 is shown in Figure 13 and Figure 14. The features extracted from the second data set are more decisive, problem-specific, and not affected by the huge shape/size difference. The input normal and defective images’ feature maps are almost similar in size. However, the feature maps of convolutional layer 3, for defective images 440 (Figure 13) and 450 (Figure 14), are brighter than input normal image 300 (Figure 11). The model trained on such a data set would be more reliable in differentiating two classes of images and earlier detection of surface distortion problems. In this case, the enormous class imbalance was handled using data augmentation methods.

The proposed model was trained on the second balanced data set to test its performance on a more genuine, natural, and challenging problem. The model performed well and achieved an accuracy of 99.08% which is slightly less than 99.31% on imbalanced data. Apart from accuracy, the model’s recall improved from 96% on the imbalanced data set to 99% on the balanced data set. This shows that the balanced data set improved the model’s performance.

**Figure 15:**
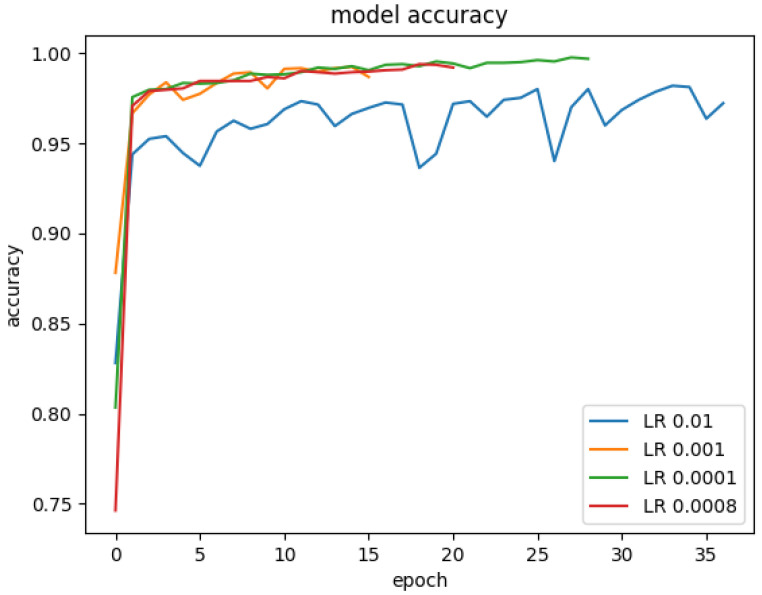
The accuracy of the model on various learning rates.

**Figure 16:**
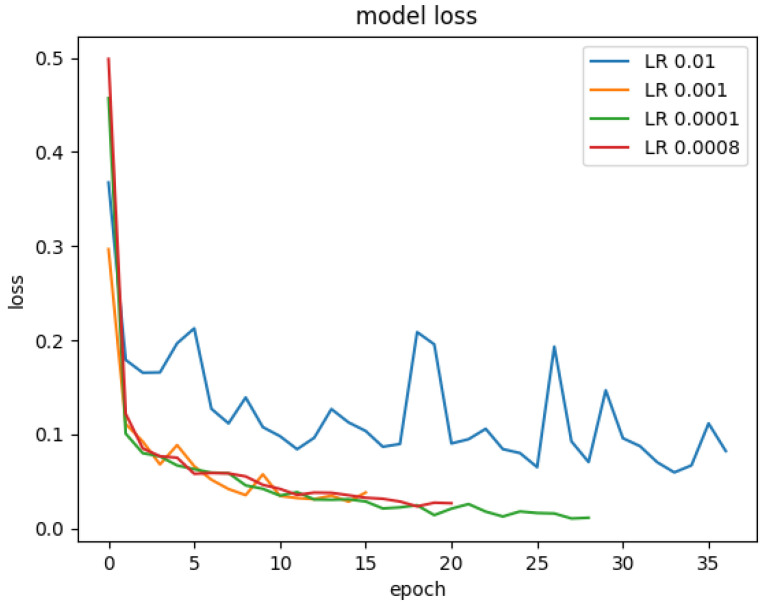
The loss of the model on various learning rates.

**Figure 17:**
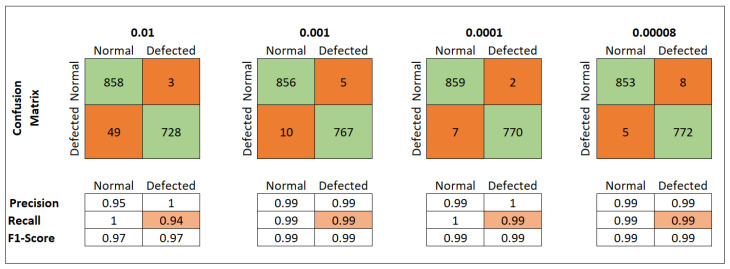
Classification report of model3 on different learning rates.

The learning rate of the optimisation algorithm plays a vital role in the model’s performance. We experimented with a range of learning rates from 0.01 to 0.00008 on our model to find the best learning rate value. Figure 15 showed the model’s accuracy on the different learning rates. The model was trained with early stopping. That is why the model has different epochs for different learning rates. Similarly, the Figure 16 shows the model’s loss when trained on various learning rate values. Experiments revealed that the model performed best with a learning rate of 0.0001. A confusion matrix and other model evaluation metrics were employed to provide an in-depth evaluation of the model for different learning rates. The model’s precision, recall, f1-score and confusion matrix on various learning rates is shown in Figure 17.

The model achieved 99.45% accuracy and recall of 99% with only 9, in total, missclassifications of the test data set.

Surface deformation defect is a multi-factor problem that cannot be avoided entirely. As the literature review suggests, there are various ways to combat it. The most prominent ones are pre-heating the substrate, increasing or decreasing the dwell time according to metal powder, and appropriate values of processing parameters such as laser power, scan speed, scan strategies and layer thickness. However, currently, there is no means to overcome or avoid surface deformation in real-time. Error-free printing of 3D objects requires a trial-and-error process to find the optimum values of the printing parameters. Even after the time-consuming process of selecting *good* values for the printing parameters has been carried out, there is still a high chance of defects occurring due to the many unexpected circumstances, such as the variability of different AM processes, printer manufacturers, and metal powders. All possible solutions to combat surface deformation presented in the literature are insufficient to determine if surface deformation has occurred in real-time. Real-time identification of surface distortion would allow the printer handler to adjust the processing parameters and avoid/limit surface deformation. This will save on the cost, time and effort of post-processing.

We successfully demonstrated the CNN’s ability to accurately identify the surface deformation problem. We experimented with a range of CNN architectures and trained and tested various models to achieve a realistic solution for surface deformation in LPBF. The final model had three convolution and pooling layers and achieved an accuracy of 99.31% on the original imbalanced data set and 99.45% on the re-sampled balanced data set. A closed-loop data-driven image-based machine learning framework will identify the surface distortion problem at an early stage. This gives the printer operators an opportunity to either rectify the problem by adjusting appropriate printing parameters or halt the printing process altogether to prevent printer damage and waste materials. Real-time monitoring of 3D metal objects will significantly reduce the post-processing cost, time, and human hours. Moreover, less post-processing will reduce the manufacturing time, and more 3D objects could be built in less time. This, in turn, increases the availability of 3D objects and assists significantly in the manufacturing shift from subtractive to additive manufacturing.

## 4. Conclusions

Surface deformation is a crucial and concerning LPBF defect that compromises the mechanical and physical properties of the 3D builds. If left unchecked, surface deformation can accumulate and damage the printer severely. There are various methods to overcome surface distortion, such as adjusting dwell time, modifying printing parameters, pre-heating the substrate and adopting an appropriate scanning strategy. However, no solution can present identify the surface deformation in real-time. We have proposed an original deep learning approach by using CNNs to identify the distortion at an early stage. We printed 13 bars with overhang geometries to create surface deformation naturally and captured powder-bed images for our ML solution. Our model is 99.3% accurate in identifying surface deformation images from normal images. The model is further tested on an improved, challenging, complex and more realistic data set to identify distortion at an earlier stage in the build process. Data augmentation, early stopping, learning rate variation and various model evaluation metrics were employed to fine-tune the model. Our model is 99.45% accurate on a re-sampled, balanced data set to identify the early signs of surface deformation. Experiments show that a balanced data set helps generalised and unbiased model learning. The proposed solution will reduce the post-processing requirement and cut the production cost of 3D builds. Moreover, it will help the printer operators adjust the printing parameters to control the damage or halt the process altogether to conserve time, material and cost.

## Figures and Tables

**Table 1:** Number of defected and non-defected images in different data sets.

Data Sets	Normal Layer Numbers	Defected Layer Numbers	Normal Images	Defected Images	Total Images
Original imbalanced data set	26 to 439	440 to 511	414	72	486
Re-sampled balanced data set	201 to 439	440 to 453	239	14	253

**Table 2:** Parameters of data augmentation of surface deformation images.

Variable	Value
Horizontal Flip	True
Vertical Flip	True
Width Shift Range	0.2
Height Shift Range	0.1
Rotation Angle	10
Fill Mode	Nearest

**Table 3:** Hyper-parameters and parameters in Convolutional Neural Networks.

Layers	Parameters	Hyper-Parameters
Convolution Layer	Kernels	Kernel Size, Number of Kernels, Stride, Padding, Activation Function.
Pooling Layer	None	Pooling Method, Filter Size, Stride, Padding
Fully Connected Layer	Weights	Number of Units, Activation Function
Others	None	Model Architecture, Optimiser Algorithm, Learning Rate, Loss Function, Mini-batch Size, Epochs, Regularisation, Weight Initialisation, Data Splitting

**Table 4:** Different Models Architectures of CNN.

Models	Layer	Parameters	Trainable Parameters
Model1	Convo1	Filters = 64, Kernel Size = 2, Padding = same, Activation = Relu	1,475,842
	Max-Pool	Stride = 10	
	Dense	Units = 320, Activation = Relu	
Model2	Convo1	Filters = 64, Kernel size = 3, Padding = same, Activation = Relu	230,730
	Max-Pool	Stride = 10	
	Convo2	Filters = 64, Kernel size = 2, Padding = same, Activation = Relu	
	Max-Pool	Stride = 4	
	Dense1	Units = 320, Activation = Relu	
	Dense2	Units = 280, Activation = Relu	
Model3	Convo1	Filters = 64, Kernel Size = 5, Padding = same, Activation = Relu	136,538
	Max-Pool	Stride = 10	
	Convo2	Filters = 48, Kernel Size = 3, Padding = same, Activation = Relu	
	Max-Pool	Stride = 4	
	Convo3	Filters = 32, Kernel Size = 2, Padding = same, Activation = Relu	
	Max-Pool	Stride = 4	
	Dense1	Units = 320, Activation = Relu	
	Dense2	Units = 280, Activation = Relu	

**Table 5:** Training and testing data sets.

Data Sets	Layer Images	Crop Images	Training Set		Testing Set	
			Normal	Defected	Normal	Defected
Original imbalanced data set	486	5832	3477	605	1491	259
Re-sampled balanced data set	253	5458	2007	1813	861	777

## Data Availability

The data is available from the corresponding author upon reasonable request.
